# Comparative genomics of clinical *Stenotrophomonas maltophilia* isolates reveals genetic diversity which correlates with colonization and persistence *in vivo*

**DOI:** 10.1099/mic.0.001408

**Published:** 2023-11-09

**Authors:** Melissa S. McDaniel, Nicholas A. Sumpter, Natalie R. Lindgren, Caitlin E. Billiot, W. Edward Swords

**Affiliations:** ^1^​ Department of Medicine, Division of Pulmonary, Allergy and Critical Care Medicine, Birmingham, AL, US; ^2^​ Gregory Fleming James Center for Cystic Fibrosis Research, Birmingham, AL, US; ^3^​ Department of Medicine, Division of Clinical Immunology and Rheumatology, Birmingham, AL, US

**Keywords:** adherence, comparative genomics, polymicrobial infection, *Pseudomonas aeruginosa*, *Stenotrophomonas maltophilia*

## Abstract

*
Stenotrophomonas maltophilia* is a Gram-negative emerging opportunistic pathogen often present in people with respiratory diseases such as cystic fibrosis (CF). People with CF (pwCF) experience lifelong polymicrobial infections of the respiratory mucosa. Our prior work showed that *Pseudomonas aeruginosa* promotes persistence of *S. maltophilia* in mouse respiratory infections. As is typical for environmental opportunistic pathogens, *S. maltophilia* has a large genome and a high degree of genetic diversity. In this study, we evaluated the genomic content of *S. maltophilia,* combining short and long read sequencing to construct nearly complete genomes of 10 clinical isolates. The genomes of these isolates were then compared with all publicly available *S. maltophilia* genome assemblies, and each isolate was then evaluated for colonization/persistence *in vivo*, both alone and in coinfection with *P. aeruginosa *. We found that while the overall genome size and GC content were fairly consistent between strains, there was considerable variability in both genome structure and gene content. Similarly, there was significant variability in *S. maltophilia* colonization and persistence in experimental mouse respiratory infections in the presence or absence of *P. aeruginosa *. Ultimately, this study gives us a greater understanding of the genomic diversity of clinical *S. maltophilia* isolates, and how this genomic diversity relates to both interactions with other pulmonary pathogens and to host disease progression. Identifying the molecular determinants of infection with *S. maltophilia* can facilitate development of novel antimicrobial strategies for a highly drug-resistant pathogen.

## Introduction

*Stenotrophomonas maltophilia* is a Gram-negative bacterium that is widely distributed in environmental reservoirs such as water and soil, and is an emerging opportunistic pathogen in people with cystic fibrosis (pwCF), particularly in the later stages of the disease [1, 2]. In the context of cystic fibrosis (CF), defects in the cystic fibrosis transmembrane conductance regulator (CFTR) result in dehydrated and viscous mucus, a decrease in mucociliary clearance, and chronic inflammation. As a result, pwCF typically experience lifelong opportunistic airway infections that are polymicrobial in nature [3–9]. Further, the delayed clearance and obstruction of airways results in a CF-specific phenomenon of micro-niche development in which regional adaptation in the colonizing microbial population over time leads to distinct populations within each bacterial species [10–14]. Adaptation to the lung environment is very important for bacterial persistence, with specific factors required for initial colonization and others highly associated with chronic infection [15]. In the case of *Pseudomonas aeruginosa *, variants typically have changes in factors associated with motility, secreted toxins and proteases [16].

*S. maltophilia* is primarily environmentally acquired, and there is little evidence that strains are effectively transmitted between pwCF. While some human-infection-associated factors have been identified, isolates must primarily rely on factors that also promote their survival in environmental situations to colonize the human lung [2, 17–21]. Our previous work described a cooperative interaction between *S. maltophilia* and *P. aeruginosa* where damage to the lung epithelium by *P. aeruginosa* contributed to the increased persistence of *S. maltophilia* [22–24]. These studies were, however, restricted to only a few strains, and given the amount of genetic diversity present in *S. maltophilia *, it is likely that genetic content plays a role in determining both the ability of an isolate to successfully infect a human host, and the amount of cooperativity that is seen with *P. aeruginosa *.

In this study, we sequenced the genomes of 10 clinical isolates of *S. maltophilia* from two different medical centres and evaluated the amount of genetic diversity present between these strains. The primary goal was to look at the impact of genetic content on cooperativity that we see between *S. maltophilia* and *P. aeruginosa *, and on the ability of *S. maltophilia* to persist in the lung on its own. We found that although strains were similar in genome size and GC content, there was significant genomic diversity between our strains, with large-scale rearrangements, insertions and deletions when compared to the genome of *S. maltophilia* K279a. Strains were grouped into previously described phylogenetic clusters for *S. maltophilia *. Clustering did not correlate with medical centre of isolation, or with survival of *S. maltophilia* in static biofilm coculture with *P. aeruginosa *. However, these clusters did correlate with both ability to persist in the murine lung alone, and the cooperative phenotype seen with *P. aeruginosa *. We further characterized the genetic diversity seen within *S. maltophilia *, which enabled us to investigate the contribution of genomic content to persistence of *S. maltophilia* strains in the lung, both in the presence and absence of *P. aeruginosa *.

## Methods

### Strains and growth conditions

*S. maltophilia* K279a is a widely used model strain originally isolated from the blood of a patient undergoing chemotherapy in 1998. Its genome has been fully annotated and sequenced [25]. This strain was provided by M. Herman (Kansas State University). *S. maltophilia* msm2, msm3, msm4 and msm6 are clinical isolates from patients at the UAB Medical Centre in Birmingham, Alabama, and were provided by W. Benjamin (University of Alabama at Birmingham) (Table 1). *S. maltophilia* ism1, ism2, ism3, ism4, ism5 and ism6 are clinical isolates from patients at UI Health Care in Iowa City, Iowa, and were provided by T. Starner (University of Wisconsin-Madison) (Table 1). *P. aeruginosa* mPA08-31 was obtained from S. Birket (University of Alabama at Birmingham). All strains were routinely cultured on Luria Bertani (LB) agar (Difco) or in LB broth. *S. maltophilia* strains were streaked for colony isolation before inoculating into LB broth and shaking overnight at 30 °C and 200 r.p.m. *P. aeruginosa* strains were streaked for colony isolation before inoculating into LB broth and shaking overnight at 37 °C and 200 r.p.m.

**Table 1.** Core genome assignment of *S. maltophilia
* Genomes of *S. maltophilia* from this study [10], and from the NCBI collection of published genomes (362) were annotated via Prokka. The core genome of *S. maltophilia* was then assigned by identifying those genes shared by at least 95 % of strains either in this study alone, or in the entire genome collection using Roary. The pangenome in each case was also calculated by adding the total number of unique genes represented by all strains.

This study only			All published genomes		
	Percentage of strains	Genes		Percentage of strains	Genes
Core genes	95–100	1209	Core genes	95–100	653
Soft core genes	94–95	0	Soft core genes	94–95	122
Shell genes	15–94	5037	Shell genes	15–94	5063
Cloud genes	0–15	8313	Cloud genes	0–15	81 205
Total genes	0–100	14 559	Total genes	0–100	87 043

### DNA extraction and sequencing

Overnight broth cultures of each strain were prepared in LB broth from a single colony to ensure a clonal population. Cultures were grown with shaking overnight at 30 °C and 200 r.p.m. Genomic DNA was extracted using the DNeasy Blood and Tissue kit (Qiagen) with manufacturer-provided modifications for Gram-negative bacteria. Preparations were quantified via nanodrop, and the quality of the preparation (size of fragments and specificity for DNA) was checked via gel electrophoresis before sequencing.

### Genomic assembly and annotation

For short read Illumina sequencing, samples were sent to SeqCenter (previously MIGS) (Pittsburgh, PA, USA). DNA sequencing was performed using an Illumina NextSeq2000 to generate paired end 151 bp reads, with ~3 000 000 reads per sample. For long read sequencing, Oxford Nanopore sequencing was also performed through SeqCenter, with ~250 000 long reads per sample, with an average length of ~10 kb each. Reads were trimmed with bcl2fastq (v. 2.20.0.445, short reads) and Porechop (v. 0.2.3_seqan2.1.1, long reads) before genome assembly and annotation. Genomes were assembled *de novo* using hybrid short and long read assembly with Unicycler (v. 0.4.8). Quality of the assembly was evaluated via QUAST (v. 5.0.2) with *S. maltophilia* K279a as the reference genome. To finish assembly, multiple contigs were combined into a continuous genome using Mauve Contig Mover in Geneious (Mauve plugin v. 1.1.1) with *S. maltophilia* K279a as a scaffold. Arranged contigs were then concatenated before moving to the annotation step. All genomes were annotated using Prokka (v. 1.14.5) and RASTtk (v. 1.3.0).

### Core genome determination and phylogenetic analysis

To evaluate the core genome of *S. maltophilia,* assembled sequences from all published *S. maltophilia* genomes were downloaded from NCBI and were annotated for gene content via Prokka (v. 1.14.5). Genome content was compared using Roary (v. 3.13.0) (parameters: -e, -n, -cd 80), and the core genome was designated as genes present in at least 80 % of the *S. maltophilia* strains. Phylogenetic relationships between strains were determined using an alignment of the core genome between strains provided by Roary. The phylogenetic tree was generated via FastTree (v. 2.1.12) and visualized in interactive tree of life (iTOL).

### Static biofilm assay

*In vitro* biofilm assays were performed according to an established micro-titre assay protocol [26] with some modifications. Biofilms were prepared from overnight broth cultures of *S. maltophilia* and *P. aeruginosa* diluted to an OD_600_ of 0.15 (~10^8^ c.f.u. ml^−1^) in LB broth. Bacterial suspensions were used to inoculate a 96-well microtitre dish (200 µl per well) and biofilms were grown at 37 °C for 12 h. Mature biofilms were washed twice with sterile PBS before plating on M9 minimal medium to select for *P. aeruginosa* and LB with gentamicin (50 µg ml^−1^) to select for *S. maltophilia *.

### Mouse respiratory infections

BALB/cJ mice (8–10 weeks old) were obtained from Jackson laboratories. Mice were anaesthetized with isoflurane and intratracheally infected with either *S. maltophilia, P. aeruginosa* or both (~10^7^ c.f.u. each in 100 µl PBS). The left lung of each mouse was harvested and homogenized in 500 µl of sterile PBS for viable plate counting. Homogenates from single-species infections were serially diluted in PBS and plated on LB to obtain viable c.f.u. counts. Homogenates from polymicrobial infections were plated on M9 minimal medium [21] for *P. aeruginosa* and LB agar containing gentamicin (50 µg ml^−1^) to enumerate *S. maltophilia *. All samples from polymicrobial infections were also plated on LB for total bacterial counts. All mouse infection protocols were approved by the UAB Institutional Animal Care and Use Committee.

### Cell culture

CF bronchial epithelial cells (CFBE41o-) cells are an immortalized human bronchial epithelial cell line homologous for the F508del mutation in CFTR [27]. Cells were routinely cultured in minimal essential medium (MEM; Corning) with 10 % FBS, and were polarized by seeding at a density of 5×10^6^ on the apical surface of transwells (0.4 µm; Corning) and growing at 37 °C for 7 days, before removing the apical media and growing for an additional 7 days at the air–liquid interface. Polarization of the epithelial membranes was confirmed via transepithelial electrical resistance measurements performed via an EVOM^2^ Volt/Ohm Meter (World Precision Instruments).

### Adherence assays

To measure the adherence of *S. maltophilia* to CFBE41o-s after prior infection with *P. aeruginosa,* cells were inoculated with ~10^6^ c.f.u. (m.o.i.=20) of *P. aeruginosa* mPA08-31 in MEM (FBS free). Bacteria were incubated on the cells for 4 h before being removed from the apical chamber. Cells were then inoculated with ~10^6^ c.f.u. (m.o.i.=20) of *S. maltophilia* (various strains) and incubated for 1 h. Cells were washed twice with sterile 1× PBS before being scraped from the transwell membrane, diluted and plated on differential medium to enumerate the bacterial burden.

### Statistical analyses

Unless otherwise noted, graphs represent sample medians ±95 % confidence intervals. For non-parametric analyses, differences between groups were analysed by the Kruskal–Wallis test with Dunn’s post-hoc comparisons corrected for multiple comparisons. For normally distributed data sets (as determined by the Shapiro–Wilk normality test) a one-way ANOVA was used with Tukey’s multiple comparisons test. Outliers were detected via the ROUT method (Q=1 %) and excluded from the analysis. All normality tests were performed on untransformed data. All statistical tests were performed using GraphPad Prism 9.

## Results

**Fig. 1.**
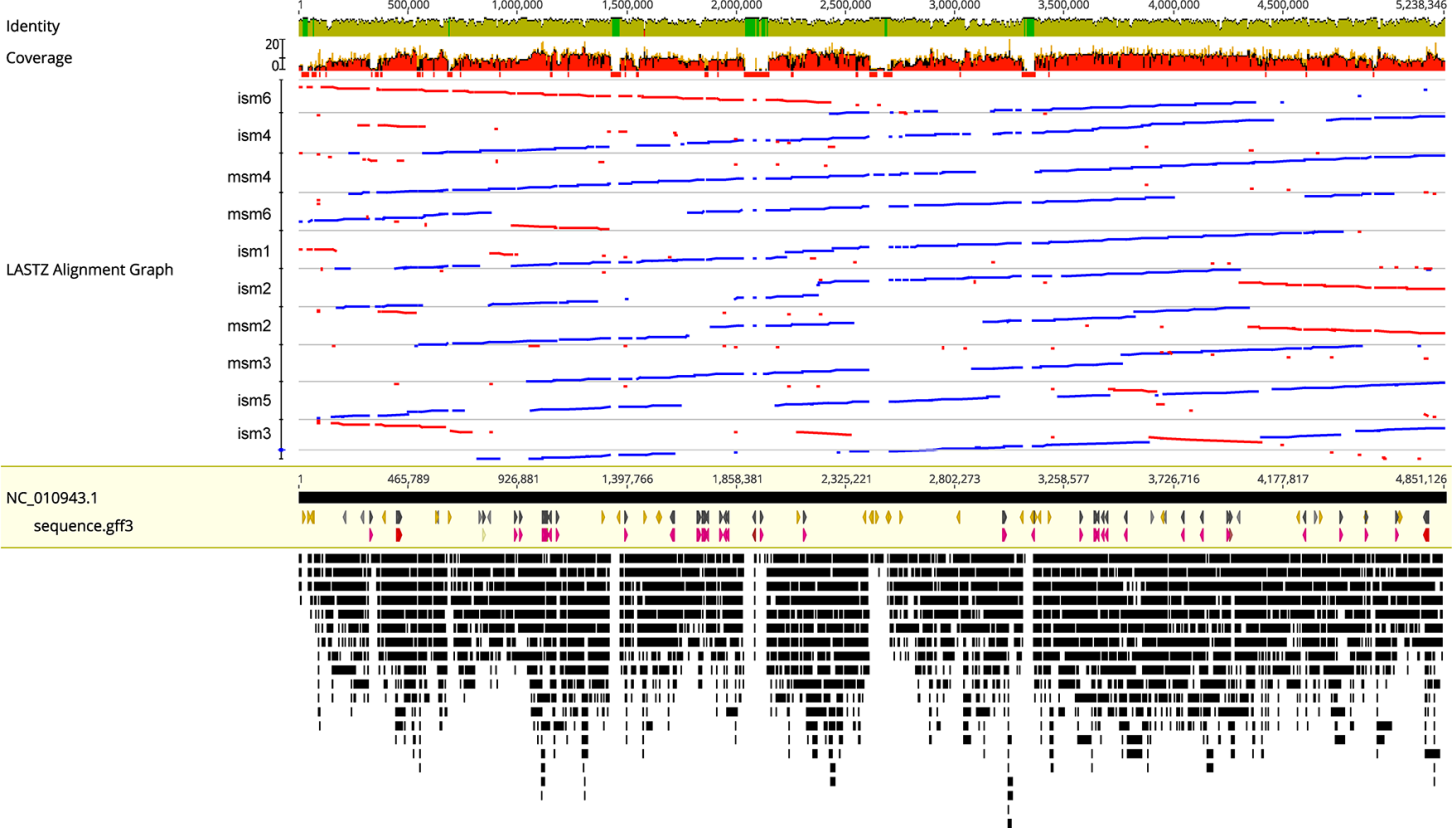
Alignment of clinical isolates to the genome of *S. maltophilia* K279a. Genomes of clinical isolates of *S. maltophilia* were sequenced using a combination of short read (Illumina) and long read (Oxford Nanopore) sequencing. Genomes were assembled via hybrid assembly using Unicycler, and contigs were rearranged according to the *S. maltophilia* K279a genome using Mauve Contig Mover. Whole genome alignments were performed using LastZ and visualized in Geneious. Blue lines represent regions of the genome in the same orientation as K279a, while red lines represent regions that are in the reverse orientation. Genomes are arranged in order of phylogeny.

To investigate the genetic diversity of *S. maltophilia* clinical isolates, we performed whole genome sequencing via a combination of short-read (Illumina) and long-read (Oxford Nanopore) sequencing on 10 clinical isolates from two medical centres. Hybrid assembly via Unicycler allowed us to assemble highly contiguous genomes for each strain, ranging from 3 to 14 total contigs and an average L_50_ of 2.1. To complete the genomes, contigs were reordered according to homology with *S. maltophilia* K279a using the Mauve Contig Mover. We found relatively little variation in both GC content and total genome length between strains, with GC content ranging from 66.2 to 67.4% and genome length ranging from ~4.0 to ~4.8 Mb (Table 2). However, alignment of the constructed genomes revealed major rearrangements in comparison to *S. maltophilia* K279a. This included insertions and deletions, inversions, and large-scale movement of genomic regions (Fig. 1).

**Table 2.** Summary statistics of genome assembly *
S. maltophilia* clinical isolates from two different medical centres were whole genome sequenced via a combination of short-read (Illumina) and long-read (Oxford Nanopore) sequencing. Genomes were assembled via hybrid assembly with Unicycler. Assembly quality was assessed using Quast, and gene annotation was performed with two commonly used annotation softwares, Prokka and RASTtk.

		Summary statistics						
	Medical centre	Isolation site	GC (%)	Length (bp)	Contig no.	CDS (Prokka)	CDS (RASTk)	L_50_
msm2	UAB	Lung	67.3	4 466 463	6	3966	4071	1
msm3	UAB	Lung	66.7	4 319 629	5	3838	3948	2
msm4	UAB	Bile	66.2	4 756 934	8	4375	4504	2
msm6	UAB	Blood	66.7	4 495 881	6	3989	4115	2
ism1	IMC	Lung	67.4	4 449 333	9	3996	4112	2
ism2	IMC	Lung	67.4	4 584 707	10	4100	4229	3
ism3	IMC	Lung	66.6	4 611 932	7	4188	4312	2
ism4	IMC	Lung	66.7	4 711 422	14	4202	4317	4
ism5	IMC	Lung	66.7	4 591 791	11	4128	4243	2
ism6	IMC	Lung	67.0	3 967 727	3	3526	3622	1

After assembling and annotating the genomes of our isolates, we identified the core genome of *S. maltophilia* via Roary. We first performed this using our sequenced strains in addition to *S. maltophilia* K279a. We found that among these strains, 2170 genes were found in at least 80 % of the strains. In total, 14 560 genes were represented in the pangenome of these 11 strains. We then performed this analysis again using every published *S. maltophilia* genome, excluding those whose 16S internal transcribed spacer (ITS) sequence identified them as other *Stenotrophomonas* species, for a total of 372 strains. In this case we found a smaller core genome shared through the entire species, with 1648 genes found in at least 80 % of the strains. As expected, the pangenome had a large number of total genes represented, with 87 043 identified (Table 1). To begin investigating the pathogenic potential of our clinical isolates, we identified antimicrobial resistance genes via ABRicate [28], and virulence genes via comparison to the virulence factor database (VFDB) [29]. Consistent with the established antimicrobial resistance (AMR) profile of *S. maltophilia,* all of our strains harbour several AMR genes, with those encoding aminoglycoside resistance and beta-lactamase production the most prevalent (Table S1, available in the online version of this article). Despite the large number of genes contained in the pangenome of our strains, these strains harboured a small selection of AMR genes in total, with most shared between strains, consistent with the previously described mechanisms of intrinsic resistance for *S. maltophilia* strains [25, 30–32]. Analysis of genomes of our sequenced strains in comparison to the VFDB identified genes with homology to many established virulence factors for other bacterial species (Table S2).

**Fig. 2.**
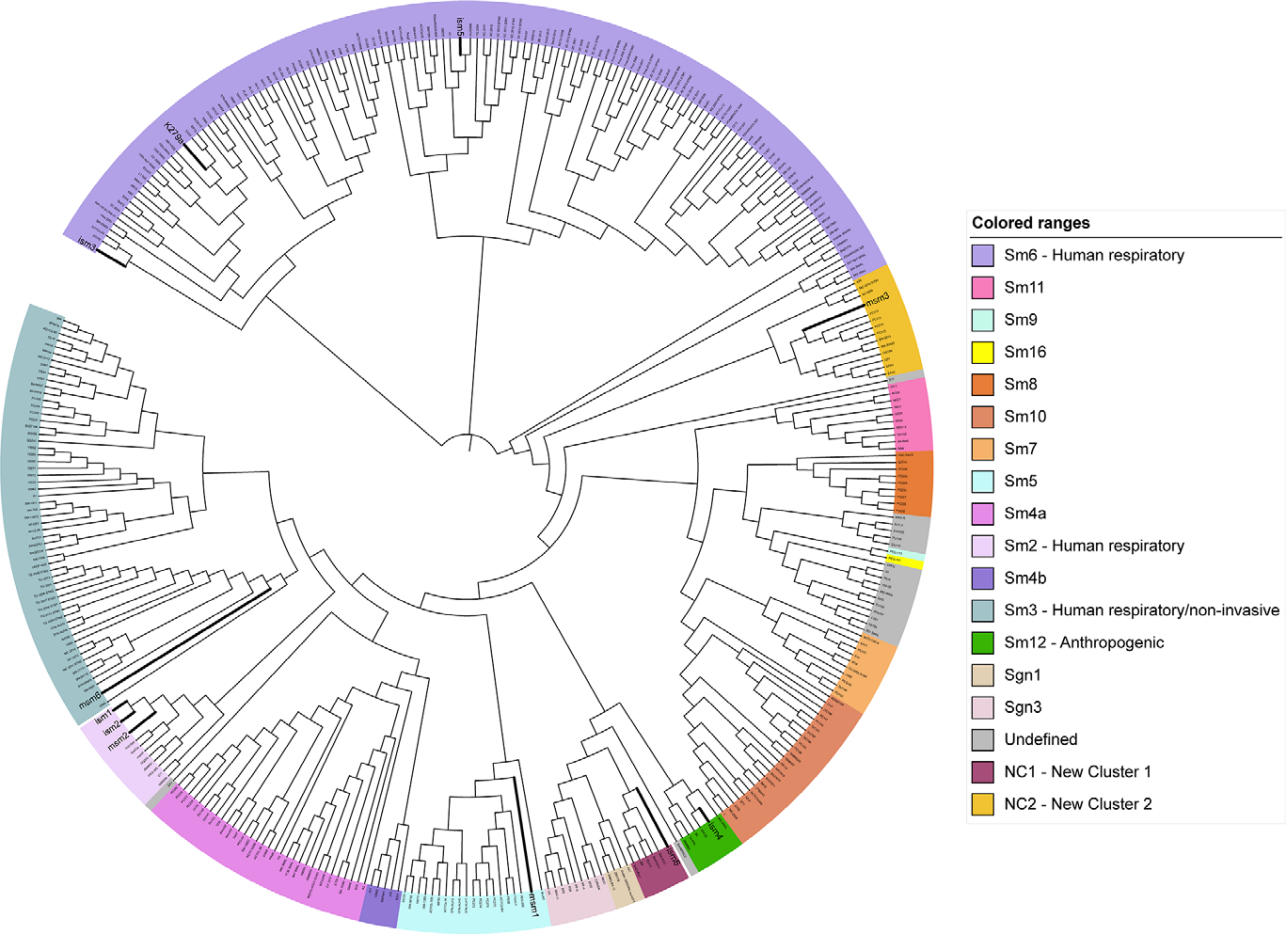
Phylogenetic tree of all published *S. maltophilia* isolates. A core genome alignment of all published isolates of *S. maltophilia *, along with those sequenced in this study, was used to reconstruct a phylogenetic tree. Clusters were coloured and labelled according to a previously published clustering scheme [10]. Branch lengths were not represented for easier visualization but can be found in Fig. S2.

Using the core genome of 1648 genes established among all 372 strains of *S. maltophilia *, we aligned all core genomes to produce an updated phylogenetic tree of *S. maltophilia* strains (Fig. 2). In previous work, an *in silico* multi-locus sequencing type (MLST) scheme had been used to identify distinct phylogenetic clusters of *S. maltophilia *, many of which correlated with site of isolation [17]. We identified most of these clusters in our tree, with 15 of the 23 clusters represented. We found that our strains localized into a total of six of these clusters, with two of our strains found in branches that have not yet been defined. *S. maltophilia* strains ism3 and ism5 clustered with K279a into Sm6, a human respiratory-associated cluster. Strains ism1, ism2 and msm2 clustered into Sm2, another human respiratory-associated cluster. Strains msm4 and msm6 clustered into Sm3, a cluster predominantly made up of both human respiratory and human non-invasive strains. Strain ism4 was in cluster Sm12, which primarily includes anthropogenic strains associated with human environmental sources. The last two strains, ism6 and msm3, did not fit into any of the predefined clusters and were therefore assigned NC1 (new cluster 1) and NC2 (new cluster 2) respectively.

**Fig. 3.**
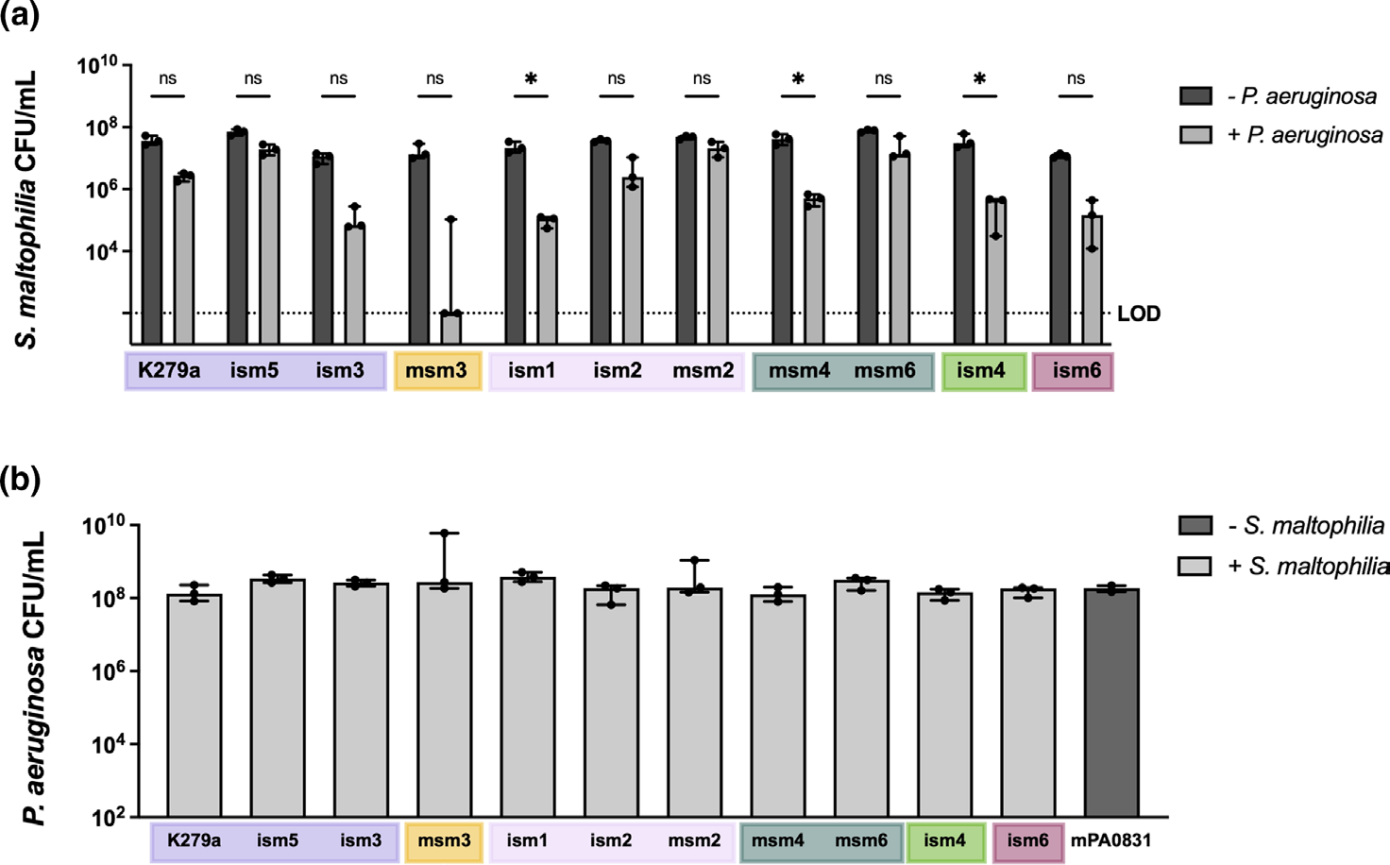
Survival of *S. maltophilia* in a static biofilm coculture with *P. aeruginosa *. Static single- and dual-species biofilms of (a) *S. maltophilia* K279a, ism1, ism2, ism3, ism4, ism5, ism6, msm2, msm3, msm4 and msm6 and (b) *P. aeruginosa* mPA0831 were seeded at ~10⁷ c.f.u. ml^–1^ of each organism in LB and grown at 30 °C for 12 h. Median ±95 % CI, *n*=3.

To assess the correlation of *S. maltophilia* phylogenetic relatedness with interaction in polymicrobial infections, we first measured the survival of clinical *S. maltophilia* isolates after coculture with *P. aeruginosa in vitro*. Although we have previously found that these organisms act cooperatively during pulmonary infections, there have been reports of antagonism between some strains during *in vitro* coculture [22, 33]. We grew each clinical isolate of *S. maltophilia* in a polymicrobial biofilm with *P. aeruginosa* mPA08-31 at 30 °C for 12 h to assess its survival. We found that *S. maltophilia* strains were inhibited by the presence of *P. aeruginosa* to varying degrees*,* but that this did not correlate with phylogenetic position (Fig. 3a). *P. aeruginosa* counts were not affected by the presence of any of the *S. maltophilia* isolates (Fig. 3b).

**Fig. 4.**
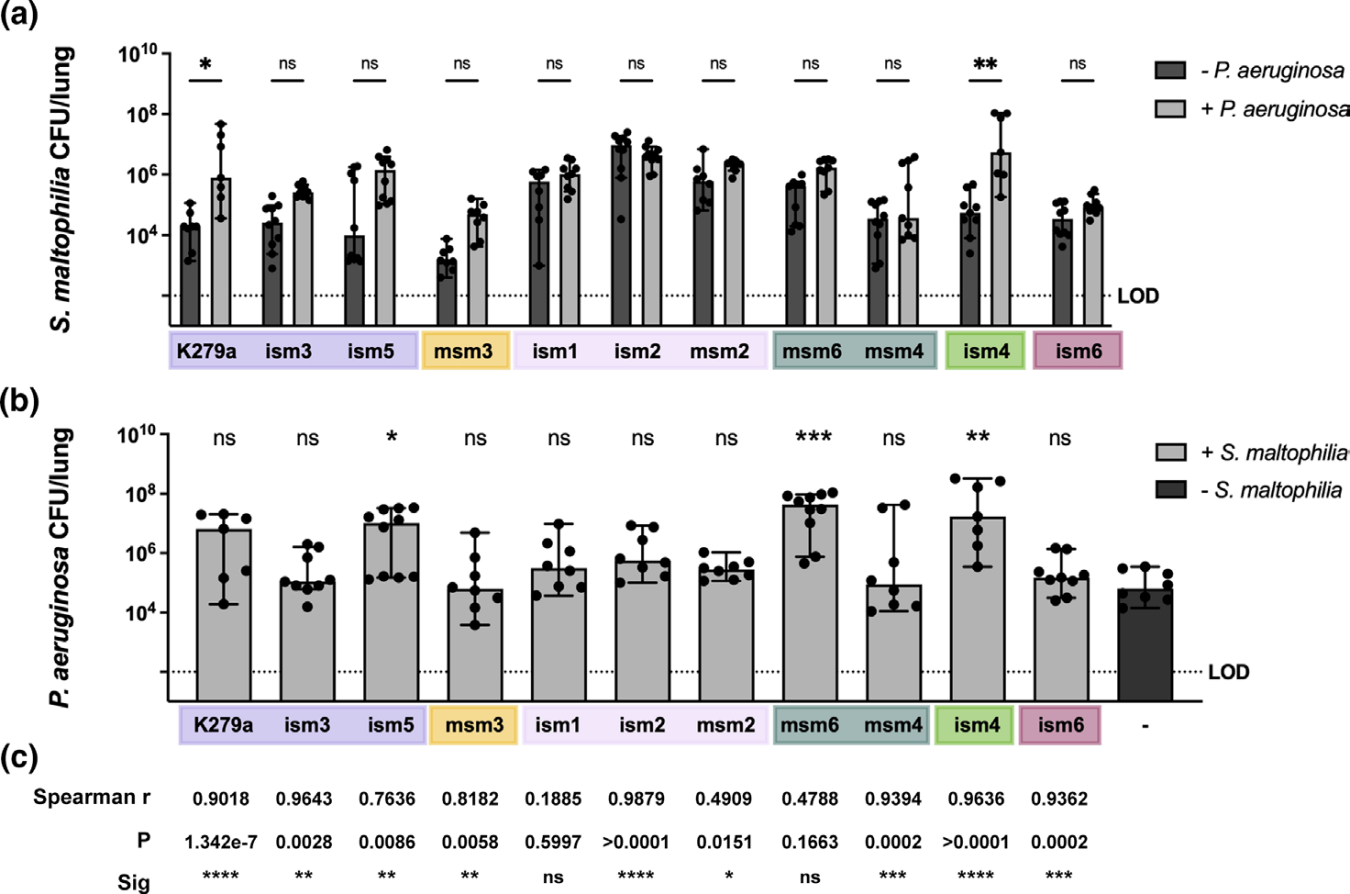
Persistence of *S. maltophilia* strains in the murine lung during polymicrobial infection with *P. aeruginosa *. BALB/cJ mice were intratracheally infected with 10^7^ c.f.u. of *S. maltophilia* K279a, ism1, ism2, ism3, ism4, ism5, ism6, msm2, msm3 or msm4 and *P. aeruginosa* mPA0831 alone and in combination. Groups were euthanized at 24 h post-infection. Bacterial load of *S. maltophilia* or *P. aeruginosa* in lung homogenate was enumerated via viable colony counting on differential medium. (**a**) *S. maltophilia* burden in the lung. Median ±95 % CI, *n*=7–9. Kruskal–Wallis test, Dunn’s post-hoc comparisons with multiple testing correction. Significant outliers were identified via ROUT and removed. (**b**) *P. aeruginosa* burden in the lung. Median ±95 % CI, *n*=7–9. Kruskal–Wallis test, Dunn’s post-hoc comparisons with multiple testing correction. Significant outliers were identified via ROUT and removed. Colours correspond to phylogenetic clusters defined in Fig. 1. (**c**) Correlations between *S. maltophilia* and *P. aeruginosa* during polymicrobial infection. These were measured using Spearman’s correlation test, with Spearman *r* and *P* values reported. *P < 0.05; **P < 0.01; ***P < 0.001; ****P<0.0001.

With these data in hand, we next performed mouse respiratory infections with each strain individually, and in polymicrobial infection with *P. aeruginosa *. Unlike in the *in vitro* biofilms, we found that phylogenetic clusters did correlate with infection outcomes, both in single-species and in polymicrobial infections. Here, we defined cooperativity as the presence of significantly higher *S. maltophilia* c.f.u. counts per lung in the presence of *P. aeruginosa* compared to single-species infection models. Only a few strains, K279a and ism4, had significantly higher *S. maltophilia* burden during polymicrobial infection, although many trended towards a higher *S. maltophilia* burden in the presence of *P. aeruginosa *. These included strains ism3 and ism5, both in the same phylogenetic cluster as K279a. One phylogenetic cluster (Sm2) showed little evidence of cooperativity, potentially due to an increased colonization/persistence of strains of *S. maltophilia* in this cluster during single-species infection (Fig. 4a).

Consistent with previous studies, we found that polymicrobial infection with *S. maltophilia* K279a provided no benefit to the persistence of *P. aeruginosa *. However, this was not the case for all the clinical isolates tested. Polymicrobial infection with several *S. maltophilia* isolates, particularly those with the highest degree of cooperativity, produced an increase in the burden of *P. aeruginosa* in the lung (Fig. 4b). In previous work on cooperativity between *S. maltophilia* and *P. aeruginosa,* we found that the bacterial burden of these organisms in the lung is highly correlated to one another. To determine if this was also the case for our clinical isolates, we measured the correlation between the bacterial burden of *S. maltophilia* and *P. aeruginosa* in the lung for each dual-species infected animal (Figs 4c and S1). In line with our previous findings, we observed that despite heterogeneity in the amount that these organisms were able to promote the bacterial burden of one another, nearly all strains showed a correlation in the bacterial burden of *S. maltophilia* and *P. aeruginosa* in the lung. Strains ism1 and msm6 were the only exceptions (Figs 4c and S1).

**Fig. 5.**
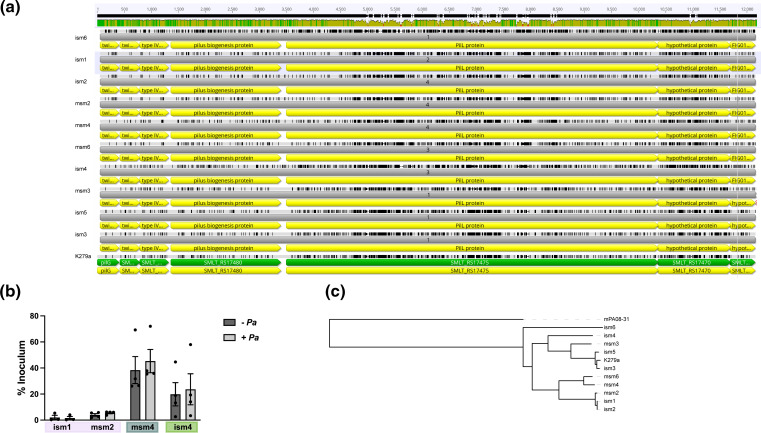
Analysis of variation in the *chpA* locus of *S. maltophilia *. The *chpA* loci of *S. maltophilia* clinical isolates were identified via annotation with RASTtk and homology to *S. maltophilia* K279a. (**a**) Sequences were aligned via clustalw on Geneious. SNPs are represented by black bars and homologous sections are represented by grey. Overall consensus is represented at the top, with green, yellow and red showing most to least similar regions between strains. (**b**) Polarized CFBEs were pre-treated with either MEM or ~10^6^ c.f.u. of *P. aeruginosa* mPA08-31 for 4 h before the addition of ~10^6^ *S. maltophilia* ism1, ism2, msm4 or ism4. Results expressed as percentage of initial *S. maltophilia* inoculum (median ±95 % CI, *n*=4 wells). (**c**) Phylogenetic relationship between the *chpA* locus of clinical strains of *S. maltophilia *, with *P. aeruginosa* mPA08-31 represented as an outgroup.

Our previous work investigating the mechanisms behind dual-species cooperativity between *S. maltophilia* and *P. aeruginosa* implicated *chpA*, the histidine kinase portion of a two-component regulatory system known to govern the type IV pilus in *P. aeruginosa *, as a determinant of the relationship between *S. maltophilia* and *P. aeruginosa* [34, 35]. We therefore decided to explore the variability in this system seen in our clinical isolates. We identified the *chpA* locus via RASTtk annotation and homology to the locus in *S. maltophilia* K279a. We found that while the complete *chpA* locus was present in all of our strains, there were many SNPs, insertions and deletions present as compared to *S. maltophilia* K279a (Fig. 5a). Adherence assays on polarized cystic fibrosis bronchial epithelial cells (CFBEs) demonstrated binding patterns that differed based on phylogeny. *S. maltophilia* strains from the Sm2 cluster (ism1 and msm2) bound poorly to polarized epithelium, which did not change following infection with *P. aeruginosa. S. maltophilia* strains from Sm3 and Sm12 clusters (msm4 and ism4 respectively) bound more efficiently to the epithelial layer, with slightly increased adherence following infection with *P. aeruginosa,* although not to a statistically significant degree (Fig. 5b). To investigate the overall relationship of our clinical strains in relation to the *chpA* locus, we constructed a phylogeny based on the alignment of the *chpA* locus and found that this phylogeny mirrors the relationships determined by the core genome (Fig. 5c).

## Discussion

In this study, we investigated the genetic diversity of clinical *S. maltophilia* strains and their ability to persist in the murine lung, alone and in combination with the important pulmonary pathogen *P. aeruginosa *. To do this, we used a combination of long and short read sequencing to assemble genomes to near completion, before comparing genetic content and assessing their position within the larger phylogeny of *S. maltophilia* strains. We found that phylogenetic position of *S. maltophilia* strains correlates with their ability to persist in the murine lung, and with the amount of cooperativity that is seen during polymicrobial infection with *P. aeruginosa *.

This study highlights the importance of using long read sequencing in combination with short reads for improved contiguity, and thus improved ability to detect structural rearrangements and insertions/deletions. Many of the insertions/deletions seen had clear evidence of either phage genes or transposon genes, explaining the large-scale rearrangements seen between strains. This also indicates that *S. maltophilia* might easily acquire genes from the environment, letting them quickly adapt, and contributing to the transition from environmental to medical contexts.

Although we were able to achieve a relatively high read depth of ~200 for both long and short reads, this was not sufficient to completely circularize the genome for all strains. This made it difficult to be certain about every structural difference that we observed between strains. Though we already had relatively high long-read sequencing depth, it is possible that increasing this further would resolve this problem. However, most of the junctions between contiguous sections occurred at repetitive regions, or those with high GC content, an established problem for a variety of sequencing methods including those used here [36–39]. This highlights the difficulty of fully assembling relatively large, repetitive and GC-rich bacterial genomes, like those of *S. maltophilia *.

Interestingly, the phylogenetic cluster that showed the least cooperativity with *P. aeruginosa* (Sm2) was able to persist in the murine lung more successfully on its own when compared to other strains. It also clustered with isolates commonly found in pulmonary infections. This was not true of the other pulmonary-associated cluster (Sm6), where tested strains showed cooperativity, although not to a statistically significant degree, with *P. aeruginosa *. It is possible that these two phylogenetic groups are representing different phases of infection, one isolated from the lung early in infection, and one that has already adapted to the lung over time. The inverse relationship between adherence to epithelial cells and persistence in the animals could be indicative of adaptation to the lung, with factors such as motility being some of the first that are lost in chronic infection. The only strain other than K279a to show a statistically significant degree of cooperativity, msm6, is from an anthropogenic cluster, again indicating human association.

We identified a large amount of genetic diversity between *S. maltophilia* strains, consistent with other studies of opportunistic pathogens that occupy an environmental niche [40–42]. Previous investigations of *S. maltophilia* genetic diversity have found similarly large amounts of genetic diversity, with no one cluster being responsible for the majority of human-related infections. Our phylogenetic tree was also able to recapitulate most of the established genetic clusters, despite the fact that previous work relied primarily on *in silico* MLST [17]. Other papers have previously established core genomes of *S. maltophilia* either by individual gene or by orthogroup [43–46]. Xu *et al*. established a core genome of 1612 genes at a stricter cutoff of 100 % with only 42 strains, as opposed to the more lenient 80 % cutoff that we used to achieve a similarly sized core genome with 372 strains. This reflects the inverse relationship between the number of strains included and the number of genes in the core genome, as shown by our smaller analysis of only our sequenced strains finding 2170 genes in the core genome vs the 1648 genes assigned to the core genome when using all 372 strains. Overall, our results are in line with previously published studies that indicate that *S. maltophilia* has an incredible amount of genetic diversity, in line with its environmental niche.

Although we were able to correlate our strains to phylogenetic clusters already associated with specific sites of origin, we are missing specific metadata associated with disease severity metrics and information on co-isolation with other organisms for the strains used in this study. Performing a similar study on a larger number of samples along with data on disease severity, patient outcomes or co-isolating organisms would allow us to dive deeper into the molecular determinants important in successful *S. maltophilia* infections, and those that govern co-infection with other pulmonary pathogens.

Taken together, the results of this study indicate that there is significant diversity within *S. maltophilia* isolates that infect humans, and that this genetic content correlates with the ability to successfully colonize the mammalian lung. It also indicates that while lung-adapted *S. maltophilia* might not need help from external sources to persist, non-lung-adapted *S. maltophilia* strains may cooperate with other pulmonary pathogens such as *P. aeruginosa* to exacerbate disease. These results prompt further study into the specific genomic content of *S. maltophilia* that contributes to worse patient outcomes and disease progression.

## Supplementary Data

Supplementary material 1

Supplementary material 2
