# Supplementary figures and images for: Comparative genomics of clinical Stenotrophomonas maltophilia isolates reveals genetic diversity which correlates with colonization and persistence in vivo
Source: Microbiology (Reading). 2023 Nov 9;169(11):001408. doi: 10.1099/mic.0.001408 (PMC10710838; doi:10.1099/mic.0.001408)

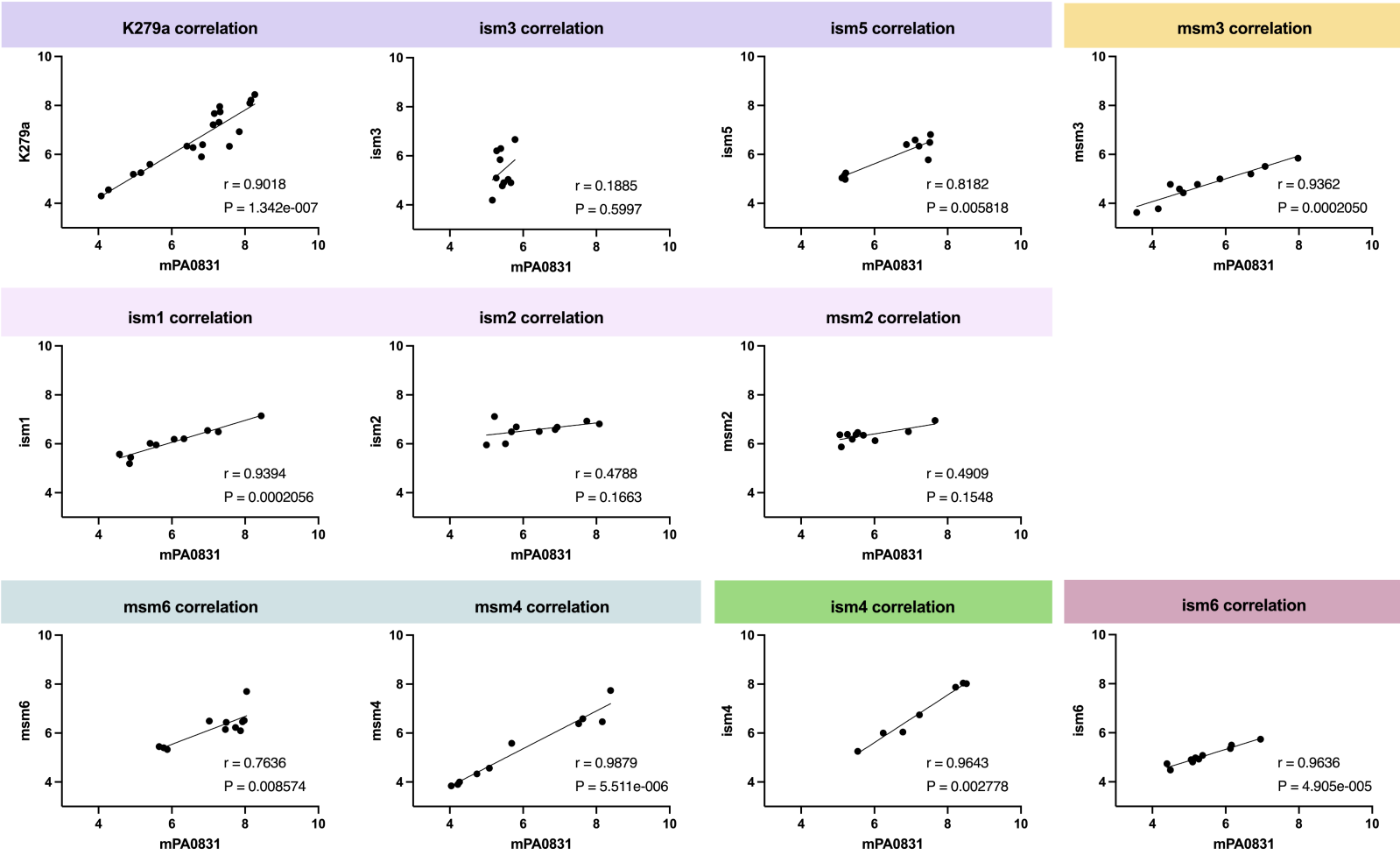

Figure S1

Supplement: Supplementary material 1 [file mic-169-1408-s001.pdf]
